# Systemic inflammation response index (SIRI) predicts mortality and cardiovascular events in maintenance hemodialysis patients: A correlational study

**DOI:** 10.1097/MD.0000000000044927

**Published:** 2025-10-10

**Authors:** Shen Jinwei, Mai Weikang, Huang Ying, Wen Yueqiang, Zhang Ying, Hong Dongxi, Huang Qian, Gao Youqun, Di Hui, Cao Xueli

**Affiliations:** a Department of Nephrology, The Second Affiliated Hospital, Guangzhou Medical University, Guangzhou, China; b Guangzhou Laboratory, Guangzhou, Guangdong, China.

**Keywords:** all-cause mortality, cardiovascular events, maintenance hemodialysis, systemic inflammation response index

## Abstract

Maintenance hemodialysis (MHD) patients generally experience a state of micro-inflammation, which increases the risk of all-cause mortality and cardiovascular events (CVEs). The systemic inflammatory response index (SIRI) is a novel inflammatory marker that reflects the body’s inflammatory state. Research has shown that SIRI not only serves as a prognostic indicator for many cancer-related diseases but also as a predictor for the occurrence of cardiovascular and cerebrovascular diseases. Currently, there is a paucity of research investigating the correlation between SIRI and all-cause mortality or the incidence of CVEs in patients undergoing MHD. This study is a retrospective cohort study, which collected 275 newly admitted MHD patients. The study population was divided into 2 groups based on the median value of SIRI. Kaplan–Meier cumulative incidence curves, multivariable logistic regression analysis, and competing risk model analysis were used to investigate the correlation between SIRI and all-cause mortality and CVE in MHD patients. Of all the patients, 275 cases of newly admitted MHD patients were recorded. Restricted cubic spline analysis revealed a linear association between the SIRI and all-cause morality as well as CVE. The Kaplan–Meier curves demonstrated differences in both all-cause mortality and CVE between the 2 groups. The competitive risk model suggested a significant difference in the cumulative incidence of all-cause mortality and CVE between the 2 groups. Compared to low SIRI group, adjusted Cox model showed that high SIRI group was associated with increased risk of all-cause mortality and CVE (all-cause mortality: odds ratio, 3.652; 95% confidence interval 1.812–7.363; *P* < .001; new-onset CVEs: odds ratio, 2.224; 95% confidence interval 1.191–4.155; *P *= .012). High SIRI levels are independent risk factors for all-cause mortality and CVEs in MHD patients.

## 1. Introduction

Chronic kidney disease (CKD) has become a global public health issue, imposing a significant economic burden on healthcare systems worldwide. According to global epidemiological studies on CKD, as of 2017, the estimated prevalence of CKD was 9.1%,^[1]^ with approximately 697.5 million CKD patients globally, and this number continues to rise. The aging population and population growth are the primary reasons for the increasing incidence of CKD.^[2]^

Numerous studies have indicated that factors such as gender, age, hypertension, diabetes, cardiovascular diseases (CVD), and obesity are all risk factors for the occurrence and progression of CKD. The incidence of CVD in CKD patients is about 3 times that of non-CKD populations, and the cardiovascular mortality rate in dialysis patients is approximately ten times that of the general population.^[3,4]^ For patients, early detection and intervention can improve disease prognosis. Therefore, the ability to predict the risk of all-cause mortality and CVE mortality in MHD patients at an early stage is of significant importance.

CVD is a complex chronic condition often associated with risk factors such as dyslipidemia, hypertension, insulin resistance, hypercoagulability, and inflammatory responses. In patients with CKD, cardiovascular events (CVEs) are the leading cause of death.^[5,6]^ A state of microinflammation is prevalent among hemodialysis (HD) patients. Studies have demonstrated that microinflammation is closely associated with the occurrence of CVD in MHD patients.^[7,8]^ The mechanism involves the microinflammatory state, characterized by an increased percentage of CD14+/CD16 + monocytes in peripheral blood. Microinflammation mediated by CD14+/CD16 + monocytes induces vascular endothelial damage, thereby elevating the risk of CVD and atherosclerosis in this population.^[9]^ Furthermore, the microinflammatory state promotes the recruitment of monocytes and lymphocytes into the arterial wall, facilitating plaque formation and accumulation. Upon plaque rupture, this can trigger CVE. High-density lipoprotein, an important antioxidant, protects endothelial cells from the effects of cytokines. However, microinflammation leads to structural and functional abnormalities in high-density lipoprotein, consequently compromising its protective effects on the vascular endothelium and increasing the risk of CVD.^[10,11]^

Research has shown that a state of micro-inflammation is commonly present in patients with CKD, and this micro-inflammatory state is closely related to the occurrence of CVD in MHD patients. Systemic inflammatory response index (SIRI), initially developed by Qi et al in 2016, is a novel micro-inflammatory marker based on the counts of peripheral blood neutrophils, monocytes, and lymphocytes. It reflects the body’s micro-inflammatory state and, compared to traditional inflammatory markers, not only better represents the immune-inflammatory status of the body but also provides more accurate predictive value for CVE. Additionally, it is easily obtainable and cost-effective.^[12,13]^

Currently, there is limited research on the correlation between SIRI and all-cause mortality and CVE in patients undergoing MHD. This study will use all-cause mortality and CVE in newly admitted MHD patients as the endpoint outcomes. Participants will be divided into high SIRI and low SIRI groups based on the median SIRI value, and relevant statistical methods will be applied to explore the relationship between SIRI levels and all-cause mortality and CVE in these 2 groups of newly admitted MHD patients.

## 2. Materials and methods

### 2.1. Subjects

**Figure 1.**
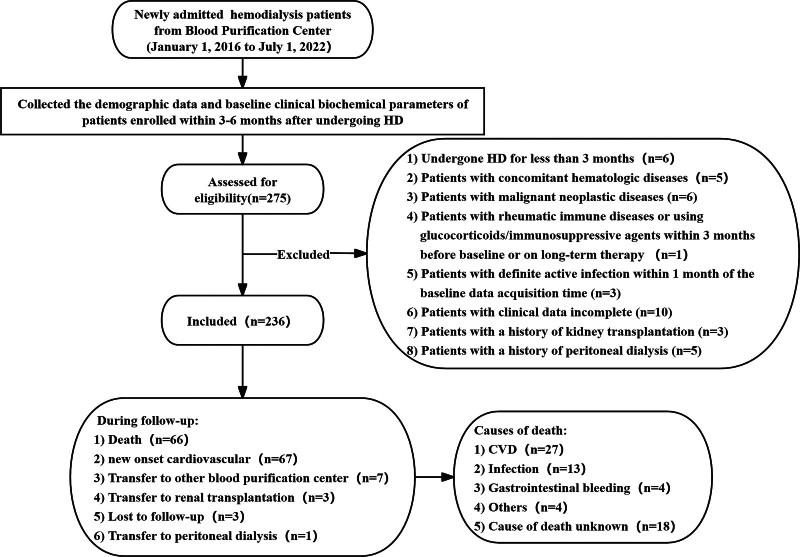
Flowchart of study patients. CVD = cardiovascular event, HD = hemodialysis, PD = peritoneal dialysis.

This study is a retrospective observational study to investigate the relationship between SIRI and all-cause mortality and CVE in newly admitted MHD patients. We retrospectively identified 275 adult patients who initiated maintenance hemodialysis (MHD) between January 1, 2016. Patients were excluded for the following reasons: the patient has undergone HD for <3 months (n = 6). Patients with concomitant hematologic diseases (n = 5), malignant neoplastic diseases (n = 6). Patients with rheumatic immune diseases (e.g., systemic lupus erythematosus or Sjögren’s syndrome) or those using glucocorticoids/immunosuppressive agents within 3 months before baseline or on long-term therapy (n = 1). Patients with definite active infection, such as pneumonia, dialysis tubing infection, and definite tissue infection, within 1 month of the baseline data acquisition time (n = 3). Patients with clinical data incomplete (n = 10). Patients with a history of kidney transplantation (n = 3), peritoneal dialysis (PD; n = 5) in the past. Based on the inclusion and exclusion criteria, a total of 236 patients were enrolled in this study (Fig. 1).

The primary endpoint of this study is all-cause mortality occurring after dialysis in newly admitted MHD patients, while the secondary endpoint is the occurrence of new CVEs. Other outcome events include transfer to other dialysis centers, transfer to PD, kidney transplantation, and loss to follow-up.

This study protocol was reviewed and approved by the Ethics Committee of The Second Affiliated Hospital of Guangzhou Medical University, approval number (IRB approval number LYZX-2025-057-01), and this research was in adherence with the Declaration of Helsinki. As this is retrospective nature of study, written informed consent was waived by the Ethics Committee of The Second Affiliated Hospital of Guangzhou Medical University.

### 2.2. Data collection

This retrospective cohort study extracted baseline demographic and biochemical data from electronic medical records of patients who initiated MHD between January 2016 and July 2022. Demographic data (e.g., age, gender, smoking/alcohol history, comorbidities [hypertension, diabetes mellitus, and CVD history], and medications [including inhibitors/angiotensin II receptor antagonists [ACEI/ARB], β-receptor antagonists and insulin]) were obtained at the initiation of HD therapy. Clinical biochemical data included C-reactive protein (CRP), white blood cell count, red blood cell count, neutrophil count, lymphocyte count, monocyte count, blood platelet count, hemoglobin, serum albumin, creatinine, uric acid, alanine aminotransferase, aspartate aminotransferase, total cholesterol, triglyceride, low-density lipoprotein cholesterol, high-density lipoprotein cholesterol, estimated glomerular filtration rate, corrected calcium, potassium and phosphorus, sp: single-pool, K: dialyzer clearance of urea, t: dialysis time, V: volume of distribution of urea (spKt/V).

CVEs are defined as acute myocardial infarction, unstable angina, acute heart failure, stroke, arrhythmias requiring treatment or life-threatening. If the patient dies because of the above causes, it is considered to be CVD death. Hypertension was recorded if the patient took antihypertensive drugs or had 2 separate blood pressure measurements ≥ 140/90 mm Hg. A history of diabetes mellitus documented in past medical history, including the history of type 1 and type 2 diabetes and other types of diabetes, is considered to be a history of diabetes. CVD diagnoses were considered for patients with one of the following conditions including cardiac arrhythmia, heart failure, coronary heart disease, cardiac arrest, or stroke.

SIRI values were computed as follows: neutrophil count × monocyte count/lymphocyte count. Body mass index was computed as weight/height^2^ (kg/m^2^). spKt/V was obtained by the BRAUN HD system. As the existing medical records were collected, written informed consent was not required.

### 2.3. Study outcome

All patients were followed up until death or CVE, transferring to PD therapy, transferring to kidney transplantation, transferring of care to other centers, lost to follow up or censoring on October 1, 2022. The primary outcome was all-cause mortality, while the secondary outcome was CVE.

### 2.4. Statistical analyses

This study divided the enrolled patients into 2 groups based on the median SIRI value for analysis. Continuous variables were described as mean ± standard deviation or median (25th–75th percentile). Categorical data were summarized using frequencies and percentages.

To assess intergroup differences, continuous variables were compared using the independent samples *t*-test (normally distributed data) or Mann–Whitney *U* test (skewed data), while categorical variables were analyzed with the Chi-square test.

The restricted cubic spline (RCS) model was constructed to fit the relationship between SIRI and all-cause mortality and CVE among MHD patients. Survival analysis was performed using the Kaplan–Meier method with Log-rank test for group comparisons. Univariate and multivariate logistic regression models were constructed to analyze independent risk factors for all-cause mortality and CVE in MHD patients.

A multivariable Cox regression model was employed to assess the impact of multiple factors on all-cause mortality and CVE in MHD patients, examining whether SIRI was independently associated with the endpoints and considering potential confounders. Hazard ratios and corresponding 95% confidence intervals (CI) were calculated. Receiver operating characteristic curves were plotted, and the area under the curve (AUC) was calculated to evaluate the predictive ability of SIRI for all-cause mortality and CVE in MHD patients.

Additionally, a competing risk model was established to examine the impact of other follow-up endpoints on all-cause mortality and CVE in newly enrolled MHD patients. All tests were two-tailed, and *P* < .05 was considered statistically significant. Missing data were filled in by the Miss Forest method. Data analysis was conducted using IBM SPSS Statistics 25 (IBM Corp., Armonk) and R software (version 4.3.1, R Foundation for Statistical Computing, Vienna, Austria).

## 3. Results

### 3.1. Participants

Baseline demographic and clinical characteristics of the cohort study were given in Table 1, categorized according to the cutoff (SIRI = 1.68) which was obtained by the median of SIRI.

**Table 1:** Baseline characteristics of patients stratified by baseline SIRI.

Variable	Cohort (n = 236)	Low SIRI group SIRI ≤ 1.68 (n = 118)	High SIRI group SIRI ≤ 1.68 (n = 118)	*P* value
Demographics				
No. of men/women	149/87	68/50	81/37	.079
Age (yr)	63.57 ± 14.42	63.47 ± 14.07	63.63 ± 14.62	.935
BMI (kg/m²)	22.18 ± 2.72	22.21 ± 2.94	22.16 ± 2.61	.896
Smoke, n (%)	43 (18.2)	16 (13.6)	27 (22.9)	.064
Drink, n (%)	13 (5.5)	7 (5.9)	6 (5.1)	.775
Comorbid				
Diabetes mellitus, n (%)	113 (47.9)	50 (42.4)	63 (53.4)	.090
Hypertension, n (%)	206 (87.3)	106 (89.8)	100 (84.7)	.241
Cardiovascular disease, n (%)	49 (20.8)	21 (17.8)	28 (23.7)	.261
Treatments				
ACEI/ARB, n (%)	142 (60.2%)	71 (60.2)	71 (60.2)	1.000
β-Blocker, n (%)	112 (47.5%)	49 (41.5)	63 (53.4)	.068
Insulin, n (%)	66 (28.0%)	25 (21.2)	41 (34.7)	.006
Statin, n (%)	99 (41.9%)	49 (41.5)	50 (42.4)	.895
Laboratory variables				
CRP (mg/L)	2.60 (0.90–6.05)	2.20 (0.70–4.30)	3.41 (1.38–8.05)	.003
White blood cell (× 10^9^/L)	6.95 ± 1.71	5.97 ± 1.36	7.43 ± 1.66	<.001
Red blood cell (× 10^9^/L)	4.21 ± 0.85	4.27 ± 0.86	4.18 ± 0.84	.387
Neutrophil (× 10^9^/L)	4.30 (3.52–5.20)	3.65 (3.01–4.28)	5.09 (4.31–6.01)	<.001
Lymphocyte (× 10^9^/L)	1.31 (1.01–1.64)	1.43 (1.16–1.71)	1.12 (0.89–1.56)	<.001
Monocyte (× 10^9^/L)	0.52 (0.40–0.65)	0.44 (0.35–0.52)	0.61 (0.51–0.74)	<.001
Blood platelet (× 10^9^/L)	203.00 (156.25–247.50)	186.5 (146.75–231.50)	213.00 (171.25–252.25)	.013
Hemoglobin (g/L)	116.00 (97.25–128.00)	118.00 (100.00–131.00)	115.50 (95.00–127.00)	.141
Serum creatinine (μmol/L)	707.00 (540.50–905.75)	726.50 (538.12–950.25)	683.50 (547.50–861.25)	.397
Serum uric acid (μmol/L)	436.38 ± 112.81	442.89 ± 116.63	433.13 ± 110.96	.511
spKt/V	1.01 (0.83–1.21)	1.07 (0.88–1.24)	0.95 (0.78–1.17)	.014
Serum albumin (g/L)	38.30 (35.23–41.20)	39.15 (35.98–2.38)	37.65 (34.70–40.40)	.006
ALT (mmol/L)	11.00 (7.00–16.00)	10.50 (7.00–16.00)	11.00 (7.00–6.00)	.006
AST (mmol/L)	13.00 (10.00–17.00)	13.00 (10.00–6.25)	14.00 (10.00–17.00)	.231
Triglycerides (mmol/L)	3.89 (3.31–4.86)	3.93 (3.32–4.94)	3.82 (3.31–4.64)	.432
Total cholesterol (mmol/L)	1.31 (0.93–2.07)	1.30 (0.94–1.97)	1.33 (0.93–2.20)	.644
LDL-C (mmol/L)	2.30 (1.78–3.01)	2.40 (1.83–3.06)	2.24 (1.76–2.92)	.584
HDL-C (mmol/L)	0.99 (0.84–1.18)	0.99 (0.86–1.25)	0.98 (0.82–1.12)	.168
eGFR (mL/min/1.73 m^2^)	6.42 (4.94–8.34)	6.05 (4.71–8.13)	6.54 (5.32–8.63)	.161
Potassium (mmol/L)	4.57 (4.02–5.12)	4.61 (4.17–5.15)	4.47 (3.90–5.11)	.128
Phosphorus (mmol/L)	1.79 (1.43–2.17)	1.74 (1.43–2.19)	1.82 (1.43–2.10)	.777
Corrected calcium (mmol/L)	2.19 (2.08–2.31)	2.18 (2.08–2.28)	2.21 (2.08–2.34)	.286
SIRI	1.68 (1.22–2.48)	1.22 (0.92–1.40)	2.48 (1.97–3.45)	<.001

ACEI/ARB = ACE inhibitors/angiotensin II receptor antagonists; β-receptor antagonists, ALT = alanine aminotransferase, AST = aspartate aminotransferase, BMI = body mass index, CRP = C-reactive protein, eGFR = estimated glomerular filtration rate, HDL-C = high density lipoprotein cholesterol, LDL-C = low density lipoprotein cholesterol, SIRI = systemic inflammation response index, spKt/V = sp, Single-Pool; K, dialyzer clearance of urea; t, dialysis time; V, volume of distribution of urea.

A total of 236 MHD patients who met the inclusion criteria were collected in this study, including 149 males (63.1%) and 87 females (36.9%), with an average age of 63.57 ± 14.42 years, 156 patients aged ≥ 60 years (66.1%), 206 patients (87.3%) with hypertension, 113 (47.9%) with diabetes mellitus, and 49 (20.8%) with cardiovascular disease history. The results showed that MHD patients were mainly elderly, and the proportion of males was greater than that of females, and hypertension was the main comorbidity of MHD patients.

The populations in different groups exhibit statistical differences in terms of CRP, white blood cell count, neutrophil count, lymphocyte count, monocyte count, blood platelet count, spKt/V, serum albumin, and, alanine aminotransferase and insulin use. Participants with lower SIRI scores tend to exhibit characteristics similar to individuals with higher lymphocyte count, spKt/V, and serum albumin levels. The median follow-up of the enrolled patients was 32 (maximum, 81) months. During the follow-up period, 66 (27.9%) deaths and 67 (28.3%) CVE were recorded, the cause of death included CVD (n = 27), infection (n = 13), gastrointestinal bleeding (n = 4), others (n = 4) and cause of death unknown (n = 18), 7 patients were transferred to other dialysis centers, 3 (1.3%) patients underwent kidney transplantation, 7 lost to follow-up, and 1 patients were transferred to PD.

### 3.2. RCS of SIRI

**Figure 2.**
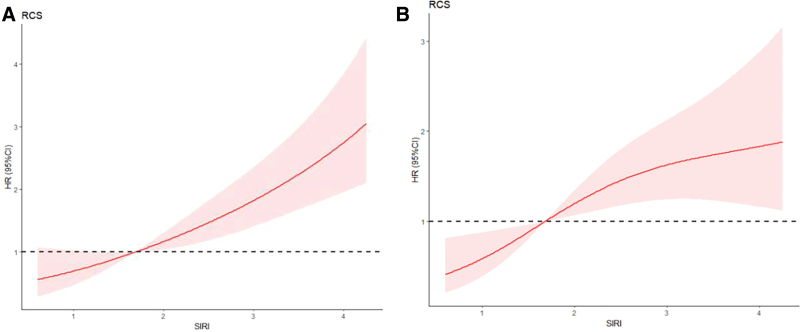
Relationship between SIRI and the all-cause mortality as well as new-onset cardiovascular events using restricted cubic spline (RCS). (A) RCS of all-cause mortality. (B) RCS of new-onset cardiovascular events. When HR = 1, there were only 1 intersection points on the RCS curve. HR = hazard ratio, RCS = restricted cubic spline, SIRI = systemic inflammation response index.

RCS indicated a linear relationship between SIRI and the all-cause mortality as well as CVE in MHD patients (Fig. 2; RCS of all-cause mortality: *P*-Overall < .0001, *P*-Nonlinear = .7328 > .05; RCS of CVE: *P*-Overall = 0.0017 < .05, *P*-Nonlinear = 0.0929 > .05). Therefore, we took median SIRI value (SIRI = 1.68) as the cutoff to divide patients into 2 groups.

### 3.3. Risk factors for higher incidence of all-cause mortality and higher

#### 3.3.1. CVE in MHD patients

According to univariable logistic regression, MHD patients with a high SIRI level at baseline correlated with a high incidence of all-cause mortality (odds ratio [OR]: 3.810, 95% CI: 2.045–7.095, *P* < .001) and CVE (OR: 2.456, 95% CI: 1.363–4.425, *P* = .003). After adjustment of the covariates with *P* < .05 in univariable logistic regression and some covariates that we judged to affect clinical outcomes based on clinical experience and previous studies, this association remained significant. (All-cause mortality: OR: 3.652, 95% CI: 1.812–7.363, *P* < .001; CVE: OR: 2.224, 95% CI: 1.191–4.155, *P* = .012; Table 2.) The results showed that the high SIRI group (SIRI > 1.68) is an independent risk factor for all-cause mortality in MHD patients (Table 2). Linear regression models were used to evaluate collinearity among the variables. The variance inflation factor was employed to quantify the severity of collinearity, with variance inflation factor < 10 indicating no significant collinearity issues between the variables. The results show that there is no multicollinearity problem in either of the 2 models (Tables S1 and S2, Supplemental Digital Content, https://links.lww.com/MD/Q269).

**Table 2:** Significant risk factors for all-cause mortality and CVEs.

Risk factors	Univariable logistic regression		Multivariable logistic regression	
	OR (95% CI)	*P*	OR (95% CI)	*P*
All-cause mortality				
Age (yr)	1.045 (1.021–1.070)	<.001	1.040 (1.013–1.068)	.004
Smoke (yes vs no)	0.532 (0.233–1.217)	.135		
Drink (yes vs no)	0.762 (0.203–2.860)	.687		
Diabetes (yes vs no)	1.874 (1.053–3.336)	.033		
Hypertension (yes vs no)	2.799 (0.937–8.357)	.065		
CVD history (yes vs no)	2.367 (1.226–4.568)	.010		
CRP (mg/L)	1.089 (1.029–1.152)	.003		
White blood cell (× 10^9^/L)	1.251 (1.055–1.483)	.010		
Red blood cell (× 10^9^/L)	0.657 (0.461–0.937)	.020		
Neutrophil (× 10^9^/L)	1.322 (1.067–1.638)	.011		
Monocyte (× 10^9^/L)	16.403 (3.361–80.055)	.001		
Hemoglobin (g/L)	0.977 (0.964–0.990)	.001	0.980 (0.964–0.995)	.010
spKt/V	0.293 (0.104–0.824)	.020		
Serum albumin (g/L)	0.840 (0.786–0.899)	<.001	0.874 (0.812–0.941)	<.001
HDL-C (mmol/L)	0.297 (0.101–0.871)	.027		
Potassium (mmol/L)	0.876 (0.621–1.236)	.451		
Phosphorus (mmol/L)	0.742 (0.441–1.248)	.261		
Corrected calcium (mmol/L)	7.844 (1.827–33.688)	.006	6.02 (1.121–32.355)	.036
ACEI/ARB (yes or no)	0.940 (0.527–1.677)	.833		
Statin (yes or no)	1.120 (0.631–1.988)	.700		
Low SIRI group vs High SIRI group	3.810 (2.045–7.095)	<.001	3.652 (1.812–7.363)	<.001
CVEs				
Age (yr)	1.027 (1.005–1.049)	.016		
Sex (female vs male)	0.588 (0.318–1.086)	.090		
Smoke (yes vs no)	1.275 (0.626–2.599)	.503		
Drink (yes vs no)	1.129 (0.335–3.798)	.845		
Diabetes (yes vs no)	3.038 (1.675–5.510)	<.001	2.250 (1.178–4.299)	.014
Hypertension (yes vs no)	4.056 (1.187–13.860)	.026	4.880 (1.352–17.622)	.016
CVD history (yes vs no)	1.834 (0.946–3.555)	.072		
White blood cell (× 10^9^/L)	1.204 (1.018–1.426)	.031		
Neutrophil (× 10^9^/L)	1.262 (1.021–1.560)	.032		
Hemoglobin (g/L)	0.990 (0.978–1.001)	.079		
spKt/V	0.515 (0.196–1.354)	.179		
Serum albumin (g/L)	0.928 (0.868–0.993)	<.001	0.928 (0.868–0.993)	.031
LDL-C (mmol/L)	1.380 (1.017–1.872)	.039		
HDL-C (mmol/L)	0.385 (0.136–1.086)	.071		
Potassium (mmol/L)	0.969 (0.692–1.358)	.856		
Phosphorus (mmol/L)	1.117 (0.689–1.811)	.652		
Corrected calcium (mmol/L)	0.802 (0.198–3.257)	.758		
ACEI/ARB (yes or no)	0.546 (0.298–1.001)	.050		
Insulin (yes or no)	2.250 (1.228–4.121)	.009		
Statin (yes or no)	1.175 (0.664–2.080)	.580		
Low SIRI group vs High SIRI group	2.456 (1.363–4.425)	.003	2.224 (1.191–4.155)	.012

ACEI/ARB = ACE inhibitors/angiotensin II receptor antagonists; β-receptor antagonists, ALT = ALANINE aminotransferase, AST = aspartate aminotransferase, CRP = C-reactive protein, CVD = cardiovascular diseases, CVE = cardiovascular event, eGFR = estimated glomerular filtration rate, HDL-C = high density lipoprotein cholesterol, LDL-C = low density lipoprotein cholesterol, OR = odds ratio, SIRI = systemic inflammation response index, spKt/V = sp, Single-Pool; K, dialyzer clearance of urea; t, dialysis time; V, volume of distribution of urea.

SIRI is associated with all-cause mortality and CVE in MHD patients. Associations of SIRI with CVE and all-cause mortality with defined models (with the lowest tertile as the reference group) are listed in Table 3. Multivariate COX regression showed that elevated SIRI was an independent risk factor for all-cause mortality as well as CVE in MHD patients after adjusting for sex, age, body mass index, diabetes, history of CVD, hypertension, CRP, hemoglobin, serum albumin, high density lipoprotein cholesterol, spKt/V, corrected calcium and ACEI/ARB. For each unit of SIRI increase, the risk of all-cause mortality increased by 1.952 times and the risk of CVE increased by 1.737 times (Table 3). The collinearity diagnosis indicates that there is no collinearity among the various variables (Table S3, Supplemental Digital Content, https://links.lww.com/MD/Q269).

**Table 3:** Relationship between SIRI and the adverse prognosis.

	HR (95%CI)	*P* value
All-cause mortality		
Unadjusted	2.855 (1.660–4.910)	<.001
Model 1	2.986 (1.724–5.172)	<.001
Model 2	2.798 (1.600–4.892)	<.001
Model 3	1.952 (1.097–3.475)	.023
New-onset CVE		
Unadjusted	2.178 (1.314–3.610)	.003
Model 1	2.215 (1.329–3.694)	.002
Model 2	2.051 (1.222–3.443)	.007
Model 3	1.737 (1.005–3.003)	.048

Reference group is low SIRI group.

Model 1: sex, age, BMI.

Model 2: Model 1 plus comorbid conditions (diabetes, history of CVD, hypertension).

Model 3: Model 2 plus CRP, hemoglobin, serum albumin, HDL-C, spKt/V, corrected calcium, ACEI/ARB.

ACEI/ARB = ACE inhibitors/angiotensin II receptor antagonists, BMI = body mass index, CRP = C-reactive protein, CVD = cardiovascular diseases, CVE = cardiovascular event, HDL-C = high density lipoprotein cholesterol, HR = hazard ratio, SIRI = systemic inflammation response index, spKt/V = sp, Single-Pool; K, dialyzer clearance of urea; t, dialysis time; V, volume of distribution of urea.

**Figure 3.**
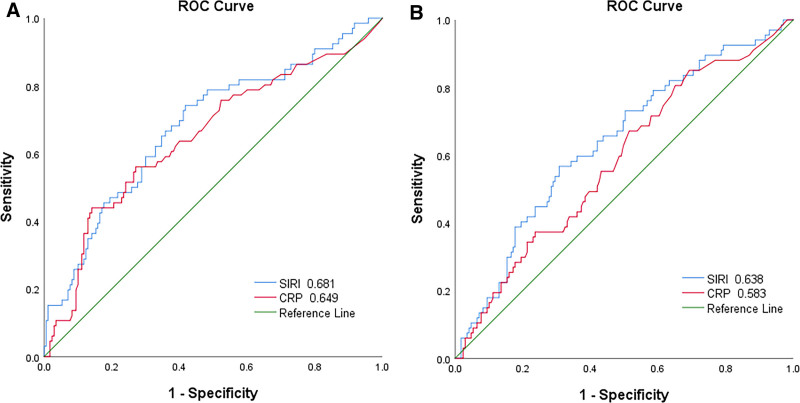
(A) ROC curves of SIRI and CRP for all-cause mortality in maintenance hemodialysis patients. The AUC value of SIRI and CRP were 0.618 and 0.649. (B) ROC curves of SIRI and CRP for CVE in maintenance hemodialysis patients. The AUC value of SIRI and CRP were 0.638 and 0.583. AUC = area under the curve, CRP = C-reactive protein, CVE = cardiovascular event, ROC = receiver operating characteristic, SIRI = systemic inflammation response index.

By measuring the AUC (Fig. 3), we compared the predictive abilities of SIRI and CRP for outcomes in MHD patients. SIRI showed good predictive capability for all-cause mortality and the CVE in MHD patients (Table 4).

**Table 4:** ROC curve analysis of inflammatory markers for all-cause mortality and new onset cardiovascular events.

	All-cause mortality			New onset cardiovascular events		
	AUC	95% CI	*P*	AUC	95%CI	*P*
SIRI	0.681	0.603–0.759	.000	0.638	0.560–0.716	.001
CRP	0.649	0.568–0.731	.000	0.583	0.503–0.663	.046

AUC = area under the curve, CRP = C-reactive protein, SIRI = systemic inflammation response index.

In this study, the primary endpoints were all-cause mortality and CVE. To account for the influence of other outcomes – transfer to other HD centers, kidney transplantation, loss to follow-up, or switch to PD – on the assessment of these endpoints, we employed a competing risks model.

**Figure 4.**
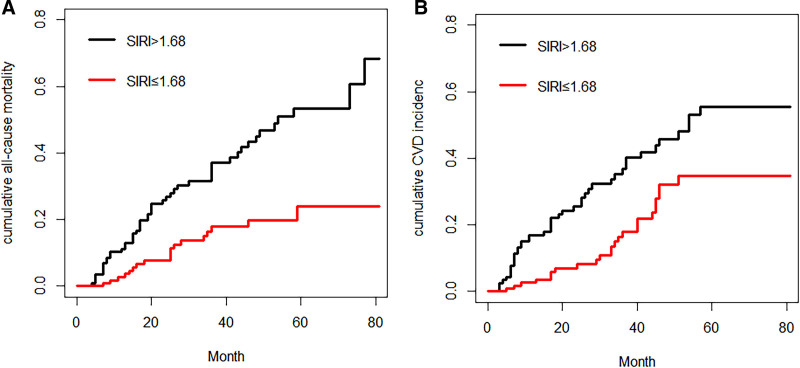
Competitive risk model. (A) All-cause mortality; (B) new-onset cardiovascular events. CVD = cardiovascular diseases, SIRI = systemic inflammation response index.

The results demonstrated that in the competing risks model for all-cause mortality among MHD patients in this study, the cumulative incidence of all-cause mortality remained significantly higher in the high SIRI group compared to the low SIRI group (Gray test 16.34, *P* < .05). Similarly, in the competing risks model for CVE among MHD patients, the cumulative incidence of CVE was also significantly higher in the high SIRI group than in the low SIRI group (Gray test 10.56, *P* < .05). There was a significant difference in the cumulative incidence function of the all-cause mortality and CVE between different SIRI groups (*P* < .05), but there were no statistically different for other endpoint events (Fig. 4).

**Figure 5.**
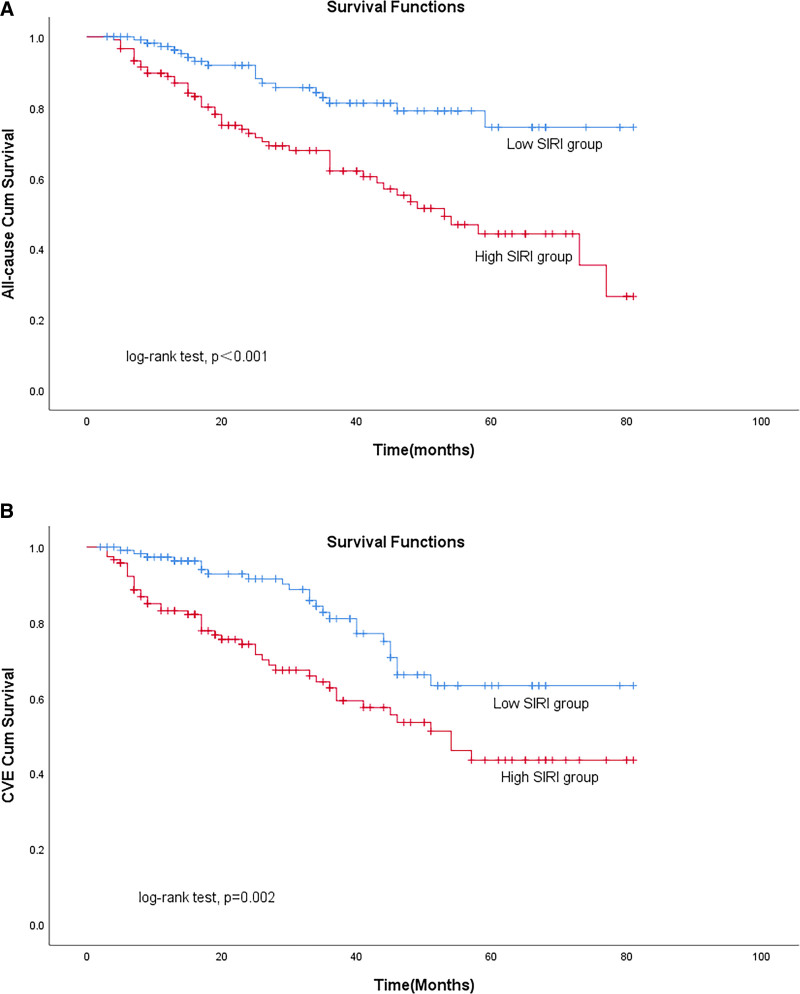
The Kaplan–Meier curves for occurrence of (A) all‐cause mortality and (B) new-onset cardiovascular events, by median value of SIRI. CVE = cardiovascular event, SIRI = systemic inflammation response index.

Kaplan–Meier cumulative incidence curve demonstrated that all-cause mortality (log-rank test *P* < .001) and the incidence of CVE (log-ranktest *P* = .002; Fig. 5) were significantly higher in the high SIRI group.

## 4. Discussion

In this study, our findings indicate that the SIRI level in MHD patients is associated with all-cause mortality and CVE. To the best of our knowledge, this is the 1st study to identify the relationship between SIRI and risk of all-cause mortality and CVE in patients on MHD.

The microinflammatory state in MHD patients refers to a condition in uremic patients who do not exhibit obvious clinical signs of systemic or local infection but have a persistent, low-level inflammatory state. This is characterized by mild elevations in inflammatory markers, including tumor necrosis factor-α, interleukin-1, interleukin-6, and CRP. Studies have shown that a microinflammatory state is commonly present in CKD patients, with an incidence of 54% to 75% among MHD patients.^[14]^ The precise mechanisms underlying the inflammatory responses in this population remain unclear; however, research suggests that several potential factors may be involved, including uremia itself, acidosis, volume overload/congestive heart failure, oxidative stress, the biocompatibility of dialysis membranes, the quality of dialysate, infections related to dialysis access, and metabolic products from gut microbiota.^[15]^ Meanwhile, the microinflammatory state is closely associated with the occurrence of CVD in MHD patients^[16,17]^.

In recent years, researchers have discovered many new inflammatory markers, such as systemic immune-inflammation index and the neutrophil to lymphocyte ratio. These markers can better reflect the body’s inflammatory state and are more accurate than traditional markers in predicting CVD. Additionally, they are easier to obtain and have lower testing costs.^[18,19]^

SIRI, initially developed by Qi et al in 2016, is a novel microinflammatory marker based on the counts of neutrophils, monocytes, and lymphocytes in peripheral blood. It reflects the body’s microinflammatory state. In that study, patients with high SIRI scores exhibited elevated serum inflammatory cytokine concentrations, shorter progression time of pancreatic cancer, and reduced survival duration. The study demonstrated that SIRI can be used to predict the prognosis of pancreatic cancer patients undergoing chemotherapy.^[20]^

In a large, 10-year prospective cohort study, elevated SIRI was found to increase the risk of stroke and all-cause mortality in the general population, even after adjusting for most potential risk factors for CVE. Moreover, the relationship remained significant even after adjusting for CRP, indicating that SIRI and CRP have different biological mechanisms in inflammation.^[21]^ Many previous studies have also shown that higher levels of SIRI are associated with the occurrence of CVD and stroke, and they affect the prognosis of the disease.^[22–26]^ In the PD population, research has indicated that higher SIRI is an independent risk factor for all-cause mortality in PD patients.^[27]^ One study demonstrated that SIRI is a novel and effective indicator for the early diagnosis of catheter-related bloodstream infections in HD patients.^[28]^ However, to the best of the authors’ knowledge, fewer studies have reported the the relationship between SIRI and all-cause mortality or CVE in patients undergoing MHD.

The mechanism by which SIRI predicts all-cause mortality and the occurrence of CVE in patients remains unclear. Inflammation is associated with the formation of atherosclerotic plaques and thrombotic events. Previous studies have indicated that neutrophils, monocytes, and lymphocytes play a key role in the inflammatory response related to atherosclerosis.^[29,30]^ Neutrophils, as 1 type of immune cell, serve as a defense line against bacterial and viral infections. Additionally, neutrophil-induced inflammation has long been considered an important factor in the pathogenesis of CVD. Activated neutrophils promote the inflammatory response in CVD through degranulation, phagocytosis, reactive oxygen species generation, and the release of neutrophil extracellular traps. Neutrophil extracellular traps are net-like structures composed of neutrophil DNA and antimicrobial proteins, including neutrophil elastase, myeloperoxidase, cathepsin G, histones, and granules. They promote thrombosis through interactions with endothelial cells and platelets and are associated with various types of CVD, such as acute coronary syndrome, deep vein thrombosis, and heart failure.^[31]^ Monocytes are released from the bone marrow into the bloodstream and are part of the mononuclear phagocyte system, playing a role in inflammation and immune responses. Circulating monocytes adhere to activated endothelial cells, infiltrate the vascular wall, and are induced by growth factors to differentiate into macrophages, which then engulf lipids and become foam cells. Lipid-rich foam cells are fundamental to the formation of atherosclerotic lesions and are an important process in this regard.^[32]^ Conversely, lymphocytes have a regulatory role in the inflammatory response and may inhibit the process of atherosclerosis. Among them, regulatory T cells are a specific subset of T cells that can prevent excessive activation of the immune system in systemic and central nervous system diseases, thereby reducing the degree of inflammation.^[33]^ Therefore, the SIRI index, composed of these 3 types of inflammatory cells, is biologically reasonable as a predictor of CVD events and all-cause mortality.

Firstly, by analyzing the data from the low SIRI group and the high SIRI group (Table 1), we found that patients in the high SIRI group had higher platelet counts (*P* = .031 by Mann–Whitney *U* test). Previous studies have shown that platelets are associated with inflammation, and both chronic and acute inflammation can alter the differentiation and number of megakaryocytes. The release of inflammatory cytokines may induce megakaryocyte rupture, leading to the rapid release of platelets as a quick replenishment for the decreased platelet count caused by inflammation.^[34]^

In addition, patients in the high SIRI group had lower serum albumin levels, which is associated with a micro-inflammatory state that can exacerbate malnutrition in MHD patients.^[35,36]^

In survival analysis, patients in the high SIRI group have a higher all-cause mortality rate and a higher incidence of CVD events. In some studies, causes of death among MHD patients primarily include cardiovascular and infectious complications, which aligns with the main causes of death for MHD patients both domestically and internationally, with CVD being the predominant cause of death for MHD patients.^[37–39]^

In the multivariate logistic regression analysis of this study, the risk of all-cause mortality and CVD events in the high SIRI group was 3.652 times and 2.224 times higher, respectively, compared to the low SIRI group. The results showed that high SIRI remained an independent risk factor for all-cause mortality and CVE in MHD patients. Chronic inflammation is associated with increased mortality in CKD patients, and persistent chronic inflammation is linked to adverse cardiovascular outcomes and poor prognosis.

Furthermore, the results of this study’s multivariate logistic regression analysis indicate that serum albumin is a protective factor for both all-cause mortality and the CVE. This may be attributed to the fact that albumin levels reflect the nutritional status of the patients, and MHD patients often experience malnutrition and muscle wasting, which can increase the risk of all-cause mortality and CVE. Maintaining good nutritional status can improve the survival outcomes of these patients. A meta-analysis on risk factors for mortality in MHD patients has also shown similar findings, indicating that low serum albumin levels increase the risk of all-cause mortality and significantly elevate the risk of cardiovascular mortality in MHD patients.^[40]^

In this study, we also established a Cox proportional hazards model for all-cause mortality and CVE in patients undergoing MHD. After adjusting for various confounding factors, the results indicated that a high SIRI group remained a risk factor for all-cause mortality and CVE in MHD patients, with risks increased by 1.952 times and 1.737 times, respectively, compared to the low SIRI group. Additionally, we plotted receiver operating characteristic curves and compared the AUC to find that SIRI had superior predictive ability for all-cause mortality and CVE in MHD patients compared to CRP. Therefore, SIRI is a valuable predictive indicator for all-cause mortality and CVE in MHD patients.

The advantage of SIRI lies in its composition, which includes monocytes, neutrophils, and lymphocytes. This not only reflects the severity of systemic inflammation but also provides a comprehensive perspective on the body’s inflammatory and immune status. Additionally, SIRI is easy to obtain and cost-effective, making it a promising candidate for widespread application. In summary, SIRI is a valuable new inflammatory marker for predicting all-cause mortality and CVEs in MHD patients.

This study has certain limitations: first, this study did not collect traditional inflammatory markers such as interleukins and procalcitonin for comparison with SIRI. Whether SIRI can better predict all-cause mortality and CVE mortality in the MHD population still requires further evaluation. Second, although this study performed multivariable adjustments in the Cox model, there may still be some unknown or uncollected risk factors that could impact all-cause mortality and CVE endpoints in MHD patients. Third, due to insufficient data, our analysis was limited to baseline SIRI values. The lack of longitudinal data and laboratory measurements at the time of endpoint events prevented us from accounting for the potential impact of dynamic SIRI changes on patient prognosis.

## 5. Conclusion

In summary, high SIRI levels are independent risk factors and excellent predictive indicators for all-cause mortality and CVE in MHD patients. Although further validation through large-scale, multicenter prospective studies is needed, SIRI, as a simple, economical, and convenient novel inflammatory marker, holds promise for clinical application and may provide valuable assistance in the early identification of adverse outcomes in MHD patients.

## Acknowledgments

We extend our highest respect and sincere gratitude to all the participants who were involved in this study.

## Author contributions

**Data curation:** Huang Qian, Gao Youqun, Di Hui, Cao Xueli.

**Formal analysis:** Zhang Ying.

**Investigation:** Hong Dongxi.

**Methodology:** Mai Weikang, Wen Yueqiang.

**Project administration:** Huang Ying.

**Writing – original draft:** Shen Jinwei.

## Supplementary Material


