# Supplementary material for: Systemic inflammation response index (SIRI) predicts mortality and cardiovascular events in maintenance hemodialysis patients: A correlational study
Source: Medicine (Baltimore). 2025 Oct 10;104(41):e44927. doi: 10.1097/MD.0000000000044927 (PMC12517796; doi:10.1097/MD.0000000000044927)
Supplement: Supplementary file 1 [file medi-104-e44927-s001.docx]

| **Supplementary Table 1. Collinearity diagnosis of the logistic regression model for all-cause mortality** | |  |
| --- | --- | --- |
| **Variable Name** | **VIF** | |
| Age (years) | 1.385 | |
| Smoke (yes vs no) | 1.230 | |
| Drink (yes vs no) | 1.200 | |
| Diabetes (yes vs no) | 1.353 | |
| Hypertension (yes vs no) | 1.187 | |
| CVD history (yes vs no) | 1.244 | |
| CRP (mg/L) | 1.355 | |
| White blood cell (× 10^9^/L) | 7.039 | |
| Red blood cell (× 10^9^/L) | 2.677 | |
| Neutrophil (× 10^9^/L) | 6.989 | |
| Monocyte (× 10^9^/L) | 2.111 | |
| Hemoglobin (g/L) | 2.729 | |
| spKt/V | 1.226 | |
| Serum albumin (g/L) | 1.615 | |
| HDL-C（mmol/L） | 1.172 | |
| Potassium (mmol/L) | 1.364 | |
| Phosphorus (mmol/L) | 1.310 | |
| Corrected calcium (mmol/L) | 1.216 | |
| ACEI/ARB (yes or no) | 1.069 | |
| Statin (yes or no) | 1.246 | |
| Low SIRI group VS High SIRI group | 1.981 | |

CRP: C-reactiveprotein; ACEI/ARB: ACE inhibitors/angiotensin II receptor antagonists; spKt/V: sp, Single-Pool; K, dialyzer clearance of urea; t, dialysis time; V, volume of distribution of urea; HDL-C: high density lipoprotein cholesterol; SIRI: systemic inflammation response index.

| **Supplementary Table 2. Collinearity diagnosis of the logistic regression model for CVE** | |  |
| --- | --- | --- |
| **Variable Name** | **VIF** | |
| Age (years) | 1.394 | |
| Diabetes (yes vs no) | 1.735 | |
| Hypertension (yes vs no) | 1.189 | |
| White blood cell (× 109/L) | 5.077 | |
| Neutrophil (× 109/L) | 6.011 | |
| Serum albumin (g/L) | 1.510 | |
| LDL-C（mmol/L） | 1.164 | |
| Insulin (yes or no) | 1.648 | |
| Low SIRI group VS High SIRI group | 1.673 | |
| Sex (female vs male) | 1.421 | |
| CVD history (yes vs no) | 1.220 | |
| Smoke (yes vs no) | 1.299 | |
| Drink (yes vs no) | 1.199 | |
| Hemoglobin (g/L) | 1.160 | |
| HDL-C（mmol/L） | 1.195 | |
| spKt/V | 1.380 | |
| Potassium (mmol/L) | 1.350 | |
| Phosphorus (mmol/L) | 1.316 | |
| Corrected calcium (mmol/L) | 1.227 | |
| Statin (yes or no) | 1.276 | |
| ACEI/ARB (yes or no) | 1.060 | |

CRP: C-reactiveprotein; ACEI/ARB: ACE inhibitors/angiotensin II receptor antagonists; spKt/V: sp, Single-Pool; K, dialyzer clearance of urea; t, dialysis time; V, volume of distribution of urea; LDL-C: low density lipoprotein cholesterol; HDL-C: high density lipoprotein cholesterol; SIRI: systemic inflammation response index.

|  | **Supplementary Table 3. Collinearity diagnosis of the Cox regression model for all-cause morality and CVE** | |  |
| --- | --- | --- | --- |
| **Variable Name** | | **VIF** | |
| Age (years) | | 1.296 | |
| Sex (female vs male) | | 1.268 | |
| BMI | | 1.303 | |
| Diabetes (yes vs no) | | 1.294 | |
| Hypertension (yes vs no) | | 1.162 | |
| CVD history (yes vs no) | | 1.130 | |
| CRP (mg/L) | | 1.288 | |
| Serum albumin (g/L) | | 1.535 | |
| Hemoglobin (g/L) | | 1.131 | |
| HDL-C（mmol/L） | | 1.267 | |
| spKt/V | | 1.356 | |
| Corrected calcium (mmol/L) | | 1.123 | |
| ACEI/ARB (yes or no) | | 1.032 | |
| Low SIRI group VS High SIRI group | | 1.108 | |

BMI: body mass index; CRP: C-reactiveprotein; ACEI/ARB: ACE inhibitors/angiotensin II receptor antagonists; spKt/V: sp, Single-Pool; K, dialyzer clearance of urea; t, dialysis time; V, volume of distribution of urea; LDL-C: low density lipoprotein cholesterol; HDL-C: high density lipoprotein cholesterol; SIRI: systemic inflammation response index.
